# Phage Therapy and Photodynamic Therapy: Low Environmental Impact Approaches to Inactivate Microorganisms in Fish Farming Plants

**DOI:** 10.3390/md7030268

**Published:** 2009-07-30

**Authors:** Adelaide Almeida, Ângela Cunha, Newton C.M. Gomes, Eliana Alves, Liliana Costa, Maria A.F. Faustino

**Affiliations:** 1 CESAM and Department of Biology, University of Aveiro, Campus Universitário de Santiago, 3810-193 Aveiro – Portugal; E-Mails:acunha@ua.pt (A.C.);gomesncm@ua.pt (N.C.M.G.);elianaalves@ua.pt (E.A.);lcosta@ua.pt (L.C.); 2 QOPNA and Department of Chemistry, University of Aveiro, Campus Universitário de Santiago, 3810-193 Aveiro – Portugal; E-Mail:faustino@ua.pt

**Keywords:** phage therapy, photodynamic therapy, fish farming, antibiotic resistance

## Abstract

Owing to the increasing importance of aquaculture to compensate for the progressive worldwide reduction of natural fish and to the fact that several fish farming plants often suffer from heavy financial losses due to the development of infections caused by microbial pathogens, including multidrug resistant bacteria, more environmentally-friendly strategies to control fish infections are urgently needed to make the aquaculture industry more sustainable. The aim of this review is to briefly present the typical fish farming diseases and their threats and discuss the present state of chemotherapy to inactivate microorganisms in fish farming plants as well as to examine the new environmentally friendly approaches to control fish infection namely phage therapy and photodynamic antimicrobial therapy.

## 1. Introduction

Aquaculture comprises all types of culture of aquatic animals and plants in fresh, brackish and marine environments [1]. Aquaculture has many forms, being the most frequent open net pens or cages in offshore areas and ponds and tanks in coastal or inland waters. Offshore aquaculture is often used to raise salmon and trout. Inland aquaculture is used to raise a variety of finfish such as catfish, yellowtail, tilapia and seabream. Offshore pens, cages and coastal ponds being open to the surrounding water means that the fish can potentially be infected with and/or transmit waterborne diseases to/from wild stocks. Fully enclosed or recirculating systems farming has the advantage of being land based and not having to be in close proximity to the sea.

Aquaculture provides nearly one-third of the world’s seafood supplies and is one of the fastest growing agricultural sector. Over the past ten years, aquaculture production has increased on average by 6% per year [2]. Production has increased from 8.7 million tons of fish in 1990 to 31.6 million tons in 2006 [2,3]. Fish farming plants, however, often suffer from heavy financial losses [4–6], due to the development of infections caused by microbial pathogens, including multidrug resistant bacteria that are easily transmitted through water and therefore able to infect a great variety of fish species.

### 1.1. Fish Farming Diseases

Cultured fishes are constantly threatened by microbial attacks. The main biological agents that cause water-borne diseases are bacteria, viruses, protists and helminths, oomycetes and, to a lesser extent, fungi. However, bacterial diseases are main problems in the expanding aquaculture industry [7–9]. Several fish farming plants often suffer from heavy losses owing to the frequent development of infections caused essentially by bacteria. There are two broad groups of bacteria of public health significance that contaminate fish: those naturally present in the environment-the indigenous microflora (e.g., *Photobacterium damselae, Vibrio anguillarum, V. vulnificus, Aeromonas hydrophila, Aeromonas. salmonicida*) and those introduced through environmental contamination by domestic animals excreta and/or human wastes – non-indigenous microflora (e.g., Enterobactereaceae such as *Salmonella sp.* and *Escherichia coli*) [10–16]. Although there is a rapid die-off of enteric bacteria in managed farm fish, significant numbers of those bacteria remain on the skin and in the guts of fish and can cause a health risk to consumers. Vibriosis and photobacteriosis (formerly pasteurellosis) are primarily diseases of marine and estuarine fish, both in natural and commercial production systems throughout the world, occurring only occasionally in freshwater fish. Vibriosis and photobacteriosis diseases can cause significant mortality in fish, reaching values of up to 100% in infected facilities, being currently responsible for the most outbreaks of fish farming plants. The vibriosis and photobacteriosis are caused by bacteria from the family Vibrionaceae. Vibriosis is caused by species from the genera *Photobacterium* (namely *Photobacterium damselae* subsp. *damselae*, formerly *Vibrio damselae*) and *Vibrio* (namely *V. anguillarum*, *V. vulnificus*, *V. alfinolyticus*, *V. parahaemolyticus* and *V. salmonicida*). Photobacteriosis is caused by *P. damselae* subsp. *piscicida* (formerly *Pasteurella piscicida*) that is a highly pathogenic bacterium that does not seem to have host specificity, infecting potentially an ample range of fish species [17,18]. Starting from 1990, European countries (especially Southern European countries) were confronted with this pathogen. Since then, several countries from the Mediterranean area such as France, Italy, Spain, Greece, Portugal, Turkey, Malta, Israel and Croatia, have been dealing with high mortality rates in cultured populations of seabass (*Dicentrarchus labrax*) and seabream (*Sparus aurata*) [19]. At the present, photobacteriosis continues to be a severe problem in intensive culture of different fish species in the Mediterranean area and Japan. Vibrionaceae species are also known to cause disease in humans, most often following to the consumption of contaminated aquaculture products. *Rickettsia*-like bacteria are also an important group of fish pathogens, affecting a variety of fish species from diverse geographical aquatic environments [20]. *A. salmonicida*, causative agent of furunculosis, is also a fish pathogen that is capable of infecting a wide range of host species [21]. *Edwardsiella tarda* causes mortalities of flounder [22,23]. A gliding bacteria *Cytophaga marina* (formerly *Flexibacter maritimus*) that produces extracellular toxins has been occurring in red and black sea breams, yellowtail and in flounder [24,25]. *Flexibacter* sp. had occurred also in sea urchin hatcheries [26]. *Flavobacterium psychrophilum* and more recently *Pseudomonas plecoglossicida* have been recognized as important pathogens of freshwater fish [27].

Viral diseases can cause heavy losses in production in marine aquaculture industry [6,28]. Although human infections caused by the consumption of fish appear to show a low risk because viruses causing disease in fish are not pathogenic to humans, many different viruses are known pathogens of already well-studied fishes, such as yellowtail [29], Japanese flounder [30,31] and black seabream [32]. Other viruses are still being discovered [33], such as spot syndrome viruses that now constitute a new virus family [34]. Viruses infecting commercial fish have been intensively studied and have found to encompass a wide range of viral families, including Irodovirus, Rhabdovirus, Birnavirus, Nodavirus, Reovirus, and Herpesviruses [35,36]. Some of these viruses have broad host ranges and seem to circulate between marine waters and freshwaters, making the transmission of the virus to new areas a serious threat. Two rhabdoviruses that cause infectious haematopoietic necrosis in salmonids of North America and haemorrhagic septicaemia in trout of Europe farms have been detected in other continents and have been also isolated in a broad range of other fish species [37–39].

Parasite-related food safety concerns in aquaculture are limited to a few helminth species, and the hazards are largely focused on communities where consumption of raw or inadequately cooked fish is a cultural habit [40]. The main human diseases caused by fish-borne parasites are trematodiasis, cestodiasis and nematodiasis. There is a lack of specific and quantitative information about the extent of the hazard in fish but the little information available indicates that trematode parasites are transmitted from farmed fish [40]. Parasites are also a concern because they often cause damage to fish tissue, creating an ideal location for bacterium infection to begin.

Infections caused by aquatic Oomycetes can affect eggs, fingerlings and adult fishes when they get injured mechanically or as a result of infections by other microorganisms. Such fish infections are common in freshwater and brackish water throughout the world [41,42] and are considered the second cause of infection, after bacteria, in freshwater aquaculture. Oomycetes infections caused by members of *Saprolegnia* represent a major challenge to a number of freshwater fishes [43,44]. *Saprolegnia* has a large impact on salmonids [45,46] but infects also a number of other fishes [47]. *Saprolegnia* infections in aquaculture were kept under control with malachite green, but this chemical has been banned worldwide since 2002 due to its carcinogenic and toxicological effects. Consequently, *Saprolegnia parasitica* is now, in economic terms, a very important fish pathogen, especially for catfish, salmon and trout species. *Aphanomyces invadans* is also an invasive oomycete pathogen of freshwater and estuarine fishes [48] that is the main aetiological agent of epizootic ulcerative syndrome, infecting many species of fish. *Phoma herbarum* has been associated with outbreaks of systemic mycosis in salmonid fish [49,50].

Diseases are more frequent in farmed fish than in the wild. Farmed fish live at greater density than wild fish which enhances the transference of pathogens between individuals. Overfeeding, high temperature and fast growth to cultivate fish as soon as possible in fish farming plants create unfavorable conditions. Moreover, overfeeding causes accumulation of organic wastes which are feed for microorganisms increasing the risk of disease outbreaks. Sick, moribund and dead fish increase also the risk of pathogens when they are not properly removed from the farming area, namely if water renewal rate is low. Also, opportunistic pathogens may become more aggressive in a polluted environment. It has been shown that pathogenic microorganisms are not introduced to the aquatic environment by farmed fish [51] but it has been suggested that wild fish are the major source of pathogens [51]. The frequency of sick individuals in wild stocks is usually low, but the number of infected individuals can be high because fish can be infected without being diseased. The wild infected fish can run rampant in densely packed offshore and inland aquaculture fish farms [51]. Records of offshore and inland aquaculture of fish indicate the prevalence of the same species of bacteria and viruses [35,52–55]. However, the parasite pathogenic communities of aquaculture cultured fish appeared to vary with changes in salinity and with a decline in fish richness and diversity [56]. Some species like salmon and trout, that are farmed mainly in offshore area, develop a specific pathogenic infectious salmon anaemia virus [57–59]. This virus causes an emerging infection in Atlantic Salmon at the time when the most prevalent bacteria infections were gradually brought under control [58]. The salmon anaemia virus was first detected in 1984 in Norwegian farms, appeared in Canada in 1996 and reached the Scottish salmon farms in 1998. Virus appeared in Clilean farms in 1999, in Farol Island and USA in 2001. The main reservoir for salmon anaemia virus is marine environment and the dominant transmission route is horizontal transmission [59]. Vertical transmission of salmon anaemia virus represents a new route of transmission within farmed populations of salmonids [60]. There is no evidence sufficient to link farmed fish to disease outbreaks in wild pacific salmon but evidence suggests that wild fish are the major sources of salmon anaemia virus [61]. Salmon and trout farms act also as a reservoir for sea lice, a parasite that is easily spread from one fish to another [62,63].

### 1.2. Preventive Measures in Fish Farming Plants

Disease prevention is the preferred health management and most cost-effective option with respect to aquaculture diseases. However, it is not always economically feasible to culture the animals under optimal conditions and give them the optimal feed, so the risk of infection and the need of effective biocontrol techniques always exist. Prevention and control of infections are especially difficult in fish farming conditions, due to the ubiquitous nature and the rapid spreading of such pathogens. The problem is further accentuated by several factors, such as (1) low microbiological water quality (high levels of faecal indicators of water quality) in many fish farms [16,64,65] (2) irregular and adverse environmental conditions (e.g., increased temperatures, salinity changes, oxygen depletion, high organic loads) which can act as predisposing agents for the outbreak of the disease, often by weakening the innate defense systems of the fish [54]; (3) high stocking densities (higher than that indicated by each species) used in fish farming plants decreases resistance to infection [54]; (4) different stages of the fish life cycle are susceptible to infections [66]; (5) increasing problems with resistance against antibiotics in common pathogenic bacteria and the concern about spreading of antibiotics in the environment [54]; (6) many chemotherapeutic agents that are effective against bacteria and oomycetes exhibit a low activity against endospores and zoospores [66]; (7) significant number of pathogenic spores persist on the fish epidermis even after quarantine [66]; and (8) few drugs are licensed for fisheries use [31,35,67].

#### 1.2.1. Vaccination

Although vaccination would be the ideal method for the prevention of infectious diseases, commercially available vaccines in the aquaculture field are still very limited [68–71]. Pasteurelosis and vibriosis have been controlled to a great extent through the use of vaccines [68,72]. A toxoid-enriched whole-cell vaccine against *Photobacterium damsela piscicida* was applied by bath immersion achieving 75% survival when applied to fish with 0.5–2.0 g. A vaccine against the *Vibrio spp*. has proven effective in European Salmonid aquaculture, especially when administered by injection. Prophylactic immunization for other bacterial diseases of farmed fish has been attempted with some success, namely against *Aeromonas salmonicida* and *Yersinia ruckeri* [72]. Vaccinated fishes appear to grow and survive better than their unvaccinated counterparts, however the exact nature of the immunity provided is not clear [71]. Currently, injectable polyvalent vaccines are used because some fish are vulnerable to infections by multiple bacterial strains. However, there are still no commercially available vaccines against two other important bacterial fish diseases: bacterial kidney disease and rickettsial septicaemia. Moreover, vaccination is not possible in the case of fish larvae, which generally are most susceptible to disease, because it is practically unfeasible to handle these small animals and also because it is believed that fish larvae do not have the ability to develop specific immunity [73].

The development of vaccines for fish viral diseases was rather unsuccessful for a long period. There were developed killed virus vaccines for two viral diseases, infectious pancreatic necrosis virus and infectious hematopoietic necrosis virus, and a recombinant DNA based vaccine for infectious pancreatic necrosis virus [74]. However, only the recombinant DNA produced vaccine is commercially available. The killed virus vaccines have produced unsatisfactory results due to killed virus residual virulence in the target species, virulence for other fish species, persistence in the treated fish and the fear that the virus might revert to virulence [75,76]. As happens with bacteria, the difficulty in developing anti-viral vaccines is due to the fact that these diseases occur primarily at the fish fry age and it is difficult to inject these small animals.

#### 1.2.2. Chemotherapy

Although chemotherapy has shown to be a rapid and effective method to treat or prevent infections, the more frequently used chemotherapeutic agents are often responsible for the development of drug resistant microbial strains. The regular use of artificial feeds supplemented with antibiotics in intensive and semi-intensive aquaculture systems to prevent the spread of disease and their massive use to control infections has resulted in the development of resistant strains, which have made antibiotic treatments ineffective. As microorganisms replicate rapidly, the possibility of a mutation quickly becomes predominant throughout the microbial population, which helps a microbe to survive, for instance, in the presence of an antibiotic drug. In fact, in marine environment more than 90% of bacterial strains are resistant to more than one antibiotic and 20% are resistant to five at least [77]. The horizontal transfer of resistance to human pathogens and the presence of antibiotic and of other drugs residues in aquaculture products for human consumption constitute important threats to public health, generating problems of allergy and toxicity and probably carrying out alterations of the normal human gut microflora [78]. Antibiotic-resistant microorganisms also enter into the marine water environment from human and animal sources. These bacteria are able to spread their genes in water-indigenous bacteria, which also contain resistance genes, increasing the problems associated with antibiotic resistant bacteria in the environment, namely in fish farming plants [79].

Approximately half of the world’s antibiotics are used for animals [80], as in fish farms, where antibiotics are used as growth promoters. In Asiatic countries where aquaculture is an important industry, a great variety of antibiotics, equivalent to 500–600 tones per year, are used in a prophylactic way, some on a daily basis [81]. In Thailand, 56 of the 76 farmers considered in the study of Holmstrom *et al*. used antibiotics [82]. More than ten different antibiotics, including chloramphenicol, gentamycin, trimethoprim, tiamulin, tetracyclines, quinolones and sulfonamids were used. Tendencia and Pena reported the use of a great variety of antibiotics in the Phillipines, including oxytetracycline, oxolinic acid chloramphenicol, furozolidine, nitrofurans, erythromycin and sulfonamids [83]. In South America, a broad range of antibiotics is also used in aquaculture, namely oxytetracycline, florfenicol, trimethoprim-sulfamethoxazole, sarafloxacin and enrofloxacin [84]. In Europe, chemotherapy is also widely practiced in aquaculture, but only a limited number of legislated antibiotics are allowed, such as amoxacillin, ampicillin, chloramphenicol, erythromycin, florfenicol, flumequine, oxolinic acid, oxytetracycline, nitrofurazone, sulphadiazine-trimethoprim and tetracycline [18,67,85,86]. The emergence of antibiotic resistant pathogens obligates the ban or restriction on the use of antibiotics by various producing (exporting) and consuming (importing) countries. This situation reflects the need for the development of alternative technologies to antibiotics in aquaculture to control bacterial pathogens.

Other biocides than antibiotics are also extensively used in the worldwide aquaculture industry to treat protozoal and fungal infections. The most frequently used are malachite green and formaldehyde. Malachite green is a triarylmethane dye that is highly effective against important protozoal and fungal infections [87–89]. Basically, it works as an ectoparasiticide, but it has also been used to control skin flukes and gill flukes. Although topically applied, these compounds might also be absorbed systemically and produce significant internal effects. Today it is well known that malachite green is environmentally persistent and produces a wide range of acute toxic effects on various fish species and certain mammals [90]. It causes serious public health hazards and also poses potential environmental problems. Consequently, malachite green has become in a highly controversial compound due to the risks it poses to the consumers of treated fish [91] including its effects on the immune, respiratory and reproductive systems and its genotoxic, carcinogenic and mutagenic properties [90]. Despite the use of this dye has been banned in 2002 in several countries, it is still being used in many parts of the world due to its low cost, ready availability, efficacy and lack of a proper alternative. On the other hand, there is also concern about the fate of malachite green, as its reduced form, leucomalachite green, that occur as a contaminant in aquatic and terrestrial ecosystems [92] and, as mentioned, potential human health hazards. Moreover, some bacterial isolates from diseased carp and trouts have been found to be resistant to malachite green [93]. More recently, potential alternative chemicals to malachite green, like bronopol (2-bromo-2-nitropropane-1,3-diol), chlorine dioxide, hydrogen peroxide and humic acids, were explored. Bronopol it is being used for the treatment of fish and appears to be a safe and effective replacement for malachite green in prevention of fungal-like infections [94,95]. Chlorine dioxide [96] and hydrogen peroxide [97] have also been found to control fungal-like infections in fish as effectively as malachite green. Humic acids have also been evaluated as alternative disinfectants [98].

Formaldehyde and formalin formulations are also used as disinfectants for prophylaxis of fish eggs and in first stages of larval development of fish [42,99]. Effects of sodium chloride, formalin and iodine on the hatching success of common carp eggs have been showed [99] and have been proposed as an effective treatment to reduce mortality in infected adult fish [42]. However, the use of formaldehyde has a significant environmental impact and is also suspected that to pose carcinogenic risk to mammals, causing harmful effects on human health [42]. Its use is therefore disencouraged, limited or even banned in several countries [100].

In this way, to reduce the risk of development and spreading of microbial resistances and to control fish diseases in aquaculture, alternative strategies must be developed to allow the use of reasonably cheap and more environmentally friendly methods. In this revision, we present the state of chemotherapy to inactivate microorganisms in fish farming plants as well as the new environmentally friendly approaches to control fish infection.

## 2. Phage Therapy: A Low Environmental Impact Technology as an Alternative to Antibiotics

Bacterial diseases are a major problem in the expanding aquaculture [7–9]. The increasing problems related with worldwide emergence of antibiotic resistance in common pathogenic bacteria, such as vancomycin-resistant enterococci and multidrug resistant staphylococci, and concern about spreading of antibiotics in the environment due to anthropogenic activities, come out the need to find new methods to control fish bacterial pathogens. Phage therapy represents a potentially viable alternative to antibiotics and to other antibacterial compounds to inactivate indigenous and non-indigenous pathogenic bacteria in fish farming plants.

### 2.1. Bacteriophages in the Marine Environment

Based on the new classification system proposed by Raoult and Forterre for viruses, a prokaryotic virus can be defined as a capsid-encoding organism that is composed by proteins and nucleic acids, self-assembles in a nucleocapsid that uses a ribosome-encoding prokaryotic organism for the completion of its life cycle [101].

The world of prokaryotic viruses, including the traditional bacteriophages (phages) and the viruses of Archaea, is currently in a period of renaissance, due to metagenomic sequencing advances and the isolation of diverse novel virus-host systems [102]. The resurgence of interest in prokaryotic viruses began in the mid 1990s as a consequence of the extraordinary abundance in the biosphere, especially in the marine environment, and of the unchallenged acceptance of the fact that viruses represent the greatest pool of genetic diversity on the planet [103–105]. Nowadays, phage research is as alive and full of promises as it was during its apogee of the 1960s and early 1970s [102]. Coincidentally, there was a parallel reawakening of interest in exploiting the enormous diversity of viruses that infect bacterial pathogens for phage therapy to control or prevent diseases from bacterial origins.

Viruses are, by far, the most abundant biological entities in the aquatic systems [106–110]. Their enormous abundance (around 10^10^–10^11^ particles L^−1^ of water) [36,111,112] and vast diversity still need more studies to provide the vital clues to the real function of viruses in natural ecosystems. The estimation of 10^30–31^ viruses in marine waters [36,108,113] corresponds to 10^23–25^ viral infections per second [36,114]. Most marine viruses are bacteriophages that kill bacteria [112], playing a significant role on the prokaryotic communities [36]. Viral lysis in surface waters removes 20–40% of the standing stocks of prokaryotes each day [115] and can match grazing by protists as a source of mortality of bacteria [111,112,116]. Consequently, viral lysis plays a significant role on the cycling of nutrients and organic matter [106]. In addition, viruses may influence the species composition of microbial communities [108]. They have a restricted range of host cells and, consequently, infection by a particular virus does not act on total microbial assemblage but rather on specific sub-populations.

In the marine environment most phages have dsDNA genome [112], belonging mainly (96% of the total) to Caudoviridales order (families Myoviridae, Siphoviridae and Podoviridae) [117]. The first metagenomic analysis focused on phages/viruses confirmed the dominance of dsDNA tailed phages in marine viral communities [118–120], but also metagenomic approach showed that a large number of sequences (6% of the total) correspond to ssDNA phages belonging to Microviridae family [103]. This group was previously overlooked because the amplification and cloning excluded ssDNA viruses. RNA phages are also present in the marine environment [121–126] but in a recent metagenomic analysis of coastal waters no RNA phages were detected [104]. However, Culley *et al*. showed that the marine environment is a reservoir of previously unknown RNA viruses, revealing that 98% of RNA viruses belong to positive-sense ssRNA viruses [104] and that the predominant hosts of marine RNA viruses are eukaryotes. In that study, however, most of the RNA phages were classified as unknown and maybe some of them are RNA phages, since there are only a few number of viral RNA sequences in the databases, which difficults viral diversity interpretation. Other studies also showed that RNA viruses are an important component of the marine virome [127].

Most of the bacteriophages used in phage therapy in marine environment belong to the dsDNA groups, specifically to Siphoviridae family [12,27,128–130] and rarely to Myoviridae family [12,27,128]. However, high mutation rates and short generation times of RNA phages make them a dynamic population of genetic variants that are capable of infecting multiple host species [131], which suggests that RNA phages might be important agents for phage therapy.

The study of virus-host interactions is essential in order to make possible use of bacteriophages to inactivate pathogenic bacteria in the aquatic environment. Viruses can interact with their hosts in two major and distinctive ways, the lytic and lysogenic cycles of infection and more sporadically through pseudolysogeny. In the lytic cycle the phages replicate their genome, producing new progeny viruses that are released through cell host lysis into the environment where they infect new bacterial cells. This capability to destroy bacteria is in the basis of using lytic phages as therapeutic or prophylactic agents. Phage multiplication is very rapid, they replicate exponentially as bacteria, declining when the number of bacteria decreases and disappearing when bacteria die. This behaviour contrasts with antibiotics where that decay, after application, occurs by excretion or by metabolic degradation.

A lysogenic infection occurs when the viral genome is integrated into the nucleic acid of host cell and replicates together with it, passing onto daughter cells. Prophages remain dormant within the host chromosome until the lytic cycle is induced by physical or chemical agents, such as radiation, pollutants and changes in temperature, salinity and nutrient concentration [132–135]. The expression of lytic genes following damage to the host DNA by any of the above mechanisms is a viral strategy that has evolved to ensure viral propagation when conditions for host survival are compromised [136]. Lysogeny might be a viral survival strategy to ensure periods of low host density during nutrient starvation [137,138]. Lysogenic bacteria may also gain specific advantages from their relationship with phages that improve their overall fitness. These effects may occur through process of conversion, whereby prophage genes are expressed in the lysogens, resulting in expanded metabolic capabilities, antibiotic resistance and toxin production, but usually in homoimmunity [139] that provides resistance to superinfection by the same or similar strains of phages. There are some classical data referring that *E. coli* cells containing prophage grow up quicker than nonlysogenic *E. coli* strain [140,141].

Pseudolysogeny (i.e., false lysogen) is described as a phenomenon where there is a constant production of phage in the presence of high host cell abundance [142]. The phage lysis results not in total host death, some cells are not destroyed, but rather in a state whereby a high abundance of phage coexists with exponential host growth. This might be the result of a mixture of sensitive and resistant host cells and/or a mixture of temperate and virulent phages. In pseudolysogeny infection, bacteriophage can either proceed with lytic infection or enter a dormant intracellular phase [108] but in this case, the phage genome does not integrate into host cellular replicons. Pseudolysogeny is an environmental condition in which starved bacterial cells coexist in an unstable relationship with infective viruses [143,144]. Under these conditions, host cells do not provide enough energy in order to phage entering into a true lysogenic or lytic condition.

The prevalence of lytic and lysogenic infection in the marine environment is a topic of considerable debate. Freifelder stated that more than 90% of known bacteriophages are temperate [138], but other authors [133,142,145] suggested that only around 50% of bacterial strains contained inducible prophages. Although a great percentage of phages are temperate, they are not suitable candidates for phage therapy since they may not immediately destroy bacteria.

### 2.2. Phage Therapy and Its Clinical Applications

Even though phages were discovered by the early 1920s and their infectious cycles understood by that time, the literature of the past half-century is almost silent on the possible therapeutic role of phages against infectious diseases. The poor understanding of mechanisms of bacterial pathogenesis and the nature of phage-host interactions, including lysogeny, led to a succession of badly designed and executed experiments. On the other hand, with the advent of antibiotic therapy, the use of phages became disregarded after the Second World War. In the 1970s, recovering previous enthusiasm on the application of phages to prevent and to treat human infections [146,147], studies of Smith *et al*. using *E. coli* models with mice and farm animals, showed that phages could be used for both treatment and prophylaxis against bacterial infections [148]. Thereafter, many other Polish and ex-Soviet Union study groups showed successful clinical usages of phages for drug-resistant infections in humans [146]. After this, many successful results using animal models were showed. The therapeutic efficacy of phage therapy against infectious diseases caused by *Pseudomonas aeruginosa* [149,150], *Staphylococcus aureus* (including methicillin-resistant *S. aureus*) [151,152], *E. coli* [147], *Enterococcus faecium* (including vancomycin-resistant *Enterococcus*) [153], *Streptococcus pneumoniae* [154], *Helicobacter pylori* [155], *Klebsiella pneumoniae* [156] and *Salmonella enteritidis* [157,158] has been shown in experimental animal models. With regards to human health, in the past, phage was administered in Poland and the Soviet Union orally, tropically or systemically to treat a wide variety of infections (suppurative wound, gastroenteritis, sepsis, osteomyelitis, dermatitis, emphysemas and pneumonia) in both adults and children [146]. In general the success rates were of 80–95% and neither adverse reactions nor reversible diseases were detectable. Consequently, in the Eastern Europe commercialization of phage therapy products was rapid. For instance, in the 1930s, Eli Lilly produced seven phage products for treat a range of infections, including abscesses, suppurating wounds and respiratory infections [159]. However, the evaluation of the efficacy of the phage therapy was based only on qualitative clinical assessment without details of doses and of other clinical criteria [146]. On the other hand, by 1940, the discovery of antibiotics diverted research attention from phage therapy, namely in the USA and Western Europe. However, the use of the phage therapy has persisted without interruption in Eastern Europe and phages are being commercialized by a number of companies [160]. The emergence of antibiotic-resistant bacteria has substancially enhanced the interest in phage therapy even by USA and Western Europe. Phage therapy is now ongoing phase I/II clinical trials and by 2011 it can reasonably be envisioned that phage treatments would be into phase III or in clinical use [102]. Nowadays, more than a dozen companies and universities are working on phage therapy for human, using current standards of clinical and microbiological research [161]. An *Escherichia coli* phase II trial is in preparation in order to control *E. coli* diarrhea by the Mestle Research Centre of Switzerland and no adverse reactions were detected. A successful phase II trial against antibiotic-resistant *Pseudomonas aeruginosa* ear infection was already completed in the UK by the BioControl Company. The company is now pursuing a phase III trial in the near future.

### 2.3. Phage Therapy and Its Fish Farm Application

Recent studies testify the use of bacteriophages as biocontrol agents in food [158,162–164], in plants [165], to control cyanobacterial blooms and for wastewater treatment [166]. Although phages of some fish-pathogenic bacteria have been described and characterized [151,167–173], these studies were mainly concerned with identifying bacteriophages for use in bacterial typing schemes or for the characterization of bacteriophages properties, including their potential role in virulence. However, there have been few attempts (Table 1) to use phages towards preventing bacterial infections in fish [15,27,174]. The results of these few studies using phages specific to *Lactococcus garvieae* and to *P. plecoglossicida*, pathogens of yellowtail and of ayu (*Plecoglossus altivelis*), respectively, suggest that phages could be useful for controlling bacterial infections of fish.

Nakai *et al* described the *in vivo* survival of *L. garvieae* bacteriophages and the potential of the phage for controlling experimental *L. garvieae* infection in yellowtail [15]. Phages were administered by injecting phage infected cells into spleen or intestine, to avoid fish defense, and also by phage-impregnated food. The survival rate was much higher in yellowtail that were injected with phages after challenge with *L. garvieae* (survival rate 100% of 20 yellowtail), compared with that of control fish without phage injection (survival rate 10% of 20 yellowtail). Simultaneous administration of live *L. garvieae* and phage enhanced the recovery of the phage from the fish organs. Protection was also obtained in yellowtail receiving phage-impregnated feed. Orally administered phage was detected at high plaque-forming units from the intestines and spleens of the phage-treated fish until 48 hours after administration. These results indicate that intraperitoneally or orally administered anti-*L. garvieae* phage prevented fish from experimental *L. garvieae* infection, suggesting the potential use of the phage for controlling the disease and that the use of bacterial cells as a protector or vehicle did not influence the curative effect of phage. Neutralizing antibodies were not detected in the fish sera that repeatedly received phages [15]. Following, two types of bacteriophages specific to *P. plecoglossicida*, the causative agent of bacterial hemorrhagic ascites disease in cultured ayu fish were isolated from diseased ayu and from rearing pond water [27]. Oral administration of phage-impregnated feed to ayu resulted in protection against experimental infection with *P. plecoglossicida*. After oral administration of *P. plecoglossicida*, cells of this bacterium were always detected in the kidneys of control fish that did not receive the phage treatment, while the cells quickly disappeared from the phage-treated fish. Ayu mortality was significantly lower in phage-treated fish (22.5% of mortality) than in the non-infected fish (60% of mortality). These results indicate that orally administered phage can be expected to kill bacterial cells in internal organs, as well as bacterial cells in the intestine, which means that phage therapy can be effective at the systemic infection stage. All phage isolates obtained in this study were *P. plecoglossicida* specific but not strain specific, suggesting that a single phage strain or a few phage strains could provide effective phage therapy. The fact that infection was established by oral route also suggests that the intestine is an important portal of entry for the pathogen, and the narrow host range of phage should be an advantage in phage treatment because the phage does not harm the normal intestinal microflora. Phage-resistant variants of *P. plecoglossicida*, which were induced *in vitro*, lacked virulence for ayu, and no phage-resistant variants were obtained from fish that died after phage treatment, suggesting that phage could be used to control this disease. The therapeutic effects against *P. plecoglossicida* infection in ayu, using the two previously isolated phages were also examined [174]. The mixture of the two phages exhibited the highest inhibitory activity. Mortalities of fish receiving one of the phage varied from 53.3 to 40.0%, decreasing to 20.0% when the mixture of the two phages were used, but increasing to 93.3% in the control fish receiving no phages. Their results showed the therapeutic effect of phages in natural infections. In a field trial, when phage-impregnated feed was administered to ayu in a pond where the disease occurred naturally (fish mortality about 10 Kg d^−1^ (ca. n = 900 d^−1^), daily mortality of fish decreased at a constant level (5 % per day) from days 3 to 15, reaching a steady state at about 6 Kg mortality d^−1^ (ca. n = 300 d^−1^) thereafter, corresponding to one-third reduction in relation to natural conditions. They confirm the success of phage treatment by detecting neither phage-resistant organisms nor phage-neutralizing antibodies in diseased fish or apparently healthy fish, respectively.

More recently, other studies on phage therapy have been carried out to control bacterial infections in fish farms. Imbeault *et al*. confirmed the earlier results of Park and Nakai [174], showing that bacteriophage combination could be successfully used in preventive programs on fish farms [175]. They studied the interaction between *A. salmonicida* and a bacteriophage to treat furunculosis in brook trout. Their results showed that more than one phage could infect *A. salmonicida* and that mutant resistant to one phage was sensitive to other or more phages. Resistant bacteria had a shorter generation time than the original strain and the success of their replating was very low. More than 25% of the mutants seemed to revert to the original strain phenotype after the first plating. All mutants were sensitive to three or more phages [175]. In another recent study, phage therapy was also used to control furunculosis of Atlantic salmon caused by *A. salmonicida* [176]. Fishes which were injected with bacteriophages immediately after challenge, died at a significant slower rate than those that were either not treated with phages or treated 24 hours past challenge. However, the end result (100% mortality) was not affected. Phages were isolated from fish that had succumbed to furunculosis but phages-resistant *A. salmonicida* isolates were recovered from mortalities in all the treatments. The results show that although there were no safety problems associated with the approach, furunculosis in Atlantic salmon is not really controllable by phages application. Walakira *et al*. isolated to two lytic bacteriophages specific for *Edwardsiella ictaluri* that cause enteric septicemia of catfish [177]. Each *E. ictaluri* strain tested was susceptible to phage infection with variable efficiencies but with no evidence of lysogeny and with no plaques detected on other bacterial species, demonstrating their potential use as biotherapeutic and diagnostic agents associated with enteric septicemia of catfish. A number of lytic phages of *Flavobacterium psychrophilum* that infect trout, causing the rainbow trout fry syndrome disease and cold water disease in fish farms were also isolated [178] and these phages showed broad host range on *F. psychrophilum*, suggesting that they could be used in phage therapy.

Some other studies focused in diseases caused by luminescent vibrios such as *Vibrio harveyi* and closed related bacteria as for instance *Vibrio campbelli*, *V. parahaemolyticus*, *Vibrio cholerae* and *V. vulnificus* that infect hatchery environments have only recently been reported (Table 1). Some of these bacteria also infect fish farm plants and cause serious human infections. A host range of seven phages from hatchery and creek water of aquaculture systems specific to *V. harveyi* were characterized and showed that all the phages were highly lytic against *V. harveyi*, showing different lytic spectrum for the large number of isolates tested (183 isolates) [128]. These phages lysed between 15 and 65% of the strains. Six of the seven phages have a broad lytic spectrum and could be potential candidates for biocontrol of the *V. harveyi* in aquatic systems. None of the phages were able to infect other *Vibrio* species. In another study a phage with lytic activity against all fifty *V. harveyi* tested were isolated from shrimp farm water [129]. This phage was tested both in laboratory system and in a hatchery for its potential to protected *Panaeus monodon* from luminous vibriosis. Microcosm studies with *P. monodon* larvae infected with *V. harveyi* showed that larval survival in the presence of phages was enhanced (80%) as compared with the control (25%). Treatment with bacteriophages improved larval survival and brought about decline in luminescent *V. harveyi* counts in hatchery tanks. Interestingly, the phages treatment performed much better than daily addition of antibiotics (5 mg L^−1^ oxytetracyclin and 10 mg L^−1^ kanamycin). The results showed that antibiotic treatment led to initial reduction of luminous bacteria after forty-eight hours when bacteria proliferate to about 10^6^ PFU mL^−1^ (survival of 40%) but when the water was treated with phages the luminous bacteria were not detected throughout the seventeen day study period (survival of 86% in the phage treated tank). The phages used in this study did not carried virulence genes. The results suggest that bacteriophages have potential for biocontrol of *V. harveyi* in hatchery systems [129]. Karunasagar *et al*. also isolated four lytic phages from oyster tissue and from shrimp hatchery water that lysed 55–70% of the one hundred *V. harveyi* isolates tested [130]. The bacteriophage treatment at 2 × 10^6^ PFU mL^−1^ level resulted in over 85% survival of *P. monodon* larvae. Bacteriophages used in this study are not associated with virulence of *V. harveyi* but are effective in controlling the population of these bacteria in hatchery systems. However, Chrisolite *et al*. showed that although phages of the luminescent *V. harveyi* occur in a hatchery and co-exist with *V. harveyi cells*, the outbreak of luminescent bacterial disease in the shrimp system remained [179].

### 2.4. Advantages of Phage Therapy over Chemotherapy in the Environment

There are several potential advantages of the application of phage therapy over chemotherapy in the environment. (1) Specific target, phages are usually highly specific to a single species or even strain of bacteria and therefore cause much less damage to the normal intestinal fish flora and to natural non-target bacteria. (2) Limited resistance development, bacteria will certainly develop resistance to phages too, but since phages have a higher mutation and replication rate, they can outcompete the adaptation of the bacteria and development of resistance is therefore limited. Moreover, it is comparably easier to find new phages than new antibiotics because phage co-evolving with their host bacteria, outnumbering bacteria in the environment by tenfold, makes possible the rapid isolation of new lytic phages from the environment for phage-resistant bacterial mutants. So, even if the bacteria acquire phage resistance, new mutant phage that acts lytically against these bacteria can be used against the targeted bacteria [151]. Smith *et al*. showed that infections produced by phage-resistant mutants of an enteropathogenic strain of *E. coli* and their parents could be successfully controlled with mutant phage derived from phage that had been active against the parent bacteria [180,181]. It is already possible to prepare a mixture of different strains of phages that would prevent the emergence of a resistant strain during phage treatment. On the other hand, purified phage-encoded peptidoglycan hydrolase (lysin) has been reported to be effective for the treatment of bacterial infectious diseases caused by Gram-positive bacteria such as *Streptococcus pyogenes*, *S. pneumoniae*, *Bacillus anthracis* and group B streptococci. Therefore, the solution to the problem of phage-resistant bacteria can be found. (3) Limited impact, unlike antibiotics, phages are self-replicating as well as self-limiting. They replicate exponentially as bacteria and decline when bacteria number decreases. Depending of the form of application, a single dose may be sufficient. Reports have revealed that a single treatment with phage leads to recovery in mice with infections caused by *E. coli* [182], *P. aeruginosa* [149], methicillin-resistant *S. aureus* [151], and vancomycin-resistant *E. faecium* [150,153]. (4) Regulatory approval, since phages are naturally occurring and very abundant, there may be substantially fewer problems involved in obtained regulatory approval for their use. (5) High resistance of phages to environmental conditions, phages are found within the same environment as their bacterial hosts, indicating the ability of these phages to survive in the same surrounding as their host bacteria. It is well known that phages are more resistant to environmental conditions that bacteria [183,184]. (6) Flexible, fast and inexpensive technology. The use of phages to control infections in aquatic environment as fish diseases seems to be particularly promising. As the host fish organisms live in aqueous media, the therapeutic phage can have continuous and closely physiological contact with the pathogens in a natural arrangement.

In line with the advantages stated above the use of bacteriophage therapy in aquaculture seems to be very promising. However, there is the undesirable possibility that phage particles may be removed from the circulatory system by host defence systems, perhaps by neutralization of the administered phages by antibodies. The preparation of several phage strains with different antigenicities may solve this problem. Duckworth and Gulig have suggested that phage therapy is usually completed before specific immunity develops [185]. Furthermore, Merril *et al*. developed a technique of serial passage in mice to select for phage mutants able to remain in the circulatory system, and they isolated phage mutants that circulated for long periods [186]. A second important problem is that lytic phages, as temperate ones [187–189] may act as vectors for transferring genes to the bacteria, namely genes that confer resistance to antibiotics and that encode virulence, transforming even non-pathogenic bacteria in pathogenic ones [173,190,191]. The global rate of phage-mediated genetic modification in bacteria has been estimated as being up to 20 × 10^15^ gene transfer events per second [192] However, it is well known that bacterial cell surface act as a virulence factor, but some of the surface components can also be the receptor for phage attachment, and consequently phage-resistant variants of a virulent organism would not be pathogenic. As stated by Anonymous (1983), adaptation of a pathogen and a phage in which the bacterial surface virulence determinant is the attachment site for the phage may be essential for successful phage therapy [193]. Consequently, before using phages for therapy it would be important to test whether they carry any virulence genes and whether it would be safe to use them.

## 3. Photodynamic Therapy: A New Antimicrobial Approach to Infectious Disease

The use of light to treat a disease is referred to as phototherapy [194,195] and its use goes back to the ancient civilizations where heliotherapy was used to improve certain disease conditions, and extracts of plants and seeds were used to enhance pigmentation induced by sunlight. Nowadays, phototherapy constitutes a branch of medicine and has its major applications in dermatological diseases, neonatal jaundice and more recently in the treatment of bladder, esophageal, gastric, cervical and lung cancers and some other diseases [196–198]. The term photodynamic action (photodynamische Wirkung) was introduced in 1903 by Hermann von Tappeiner based on Raab studies. These studies showed that the combined action of light and a drug (acridine orange) killed paramecia efficiently. Indeed the main characteristic of photochemotherapy is the fact that either factor alone is ineffective and only their concurrent action makes them therapeutically successful [199]. The photodynamic process comprises the action of three components: a photosensitizing agent, visible light and the presence of oxygen. The concept of photochemotherapy is based on the preferential affinity of the PS to target cells. The subsequent irradiation with visible light in the presence of oxygen induces cell damage extensive enough to ensure the inactivation of the microorganisms. It is generally accepted that cytotoxic agents such as singlet oxygen (^1^O_2_) and reactive oxygen radicals (hydrogen peroxide [H_2_O_2_], superoxide [O_2_^•−^], hydroxyl radical [−OH^•−^]) generated by the PS action which transfer part of the absorbed energy to molecular oxygen, are the species causing cell disruption [196,200–204].

### 3.1. Photosensitizers

**Figure 1:**
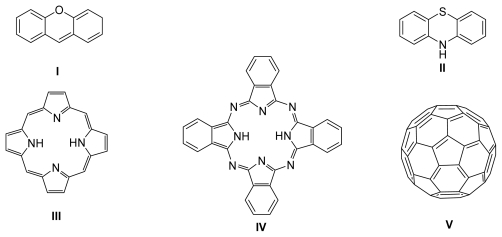
Skeletons of xanthene (I), phenothiazine (II), porphyrin (III) phthalocyanine (IV) and fullerene (V) photosensitizers.

A photosensitizer (PS) can be a natural or a synthetic compound which undergoes excitation after interaction with an appropriate radiation emitted from a light source. This gives rise to activated species (e.g., molecular oxygen) which are very reactive towards the chemical environment thus producing molecular consequences on important biological targets. For a compound to be considered for use in photodynamic treatment it must fulfill some properties which are application dependent. However, regardless of other requisites, all of the PS must have good absorption capacity at the wavelength of the spectral region where the light is emitted and show good efficiency to generate singlet oxygen [205–207]. The photosensitizing properties of organic dyes (such as rose bengal, eosin, and methylene blue), fullerenes [208], porphyrins (of natural and synthetic origin), phthalocyanines and related tetrapyrrolic macrocycles, were evaluated (Figure 1).

In photodynamic treatment of cancer, three generations of PS can be identified. The first generation comprises the Haematoporphyrin derivative (HpD) and its analogues including Photofrin^®^, the first formulation approved for cancer treatment in several countries; second generation is constituted by structurally distinct compounds with long-wavelength absorption; and the third generation comprises the above mentioned structurally distinct compounds with long-wavelength absorption, but now bound to carriers for selective accumulation in the tumors (e.g., antibodies) [205].

Porphyrins and analogues have been the most promising compounds used in photochemotherapy. Their natural occurrence and the important role in vital processes such as photosynthesis, oxygen transport and storage were responsible for decades of research devoted to the establishment of new and efficient synthetic methodologies to prepare them. New and highly efficient synthetic routes have generated many porphyrin-like compounds with unique characteristics for their uses in several other applications such as oxidative catalysis [209,210] and as biomimetic model systems of the primary processes of photosynthesis [211,212]. Presently, the interest also includes the supramolecular units, namely molecular recognition in chemical receptors and sensors [213–215], as light-harvesting devices [216–219] and as materials for advanced technologies, mainly in nanosciences [220,221]. Nevertheless, the photodynamic treatment is certainly the most promising application of porphyrin-like compounds.

The photodynamic treatment efficiency, namely in the photoinactivation of microorganisms, depends on several factors such as the presence or absence of charge, charge distribution and the presence of peripheral substituents. The porphyrin skeleton is essentially hydrophobic. This feature may be an important factor affecting the preferential accumulation in cellular hydrophobic loci since such molecules must be able to get into cells by crossing lipid membranes which brings insolubility in water and physiological fluids. To overcome this fact, adequate formulations have to be used [222–224]. However, the structural modification of the porphyrinic core can provide the required peripheral substituents (like the carbohydrate residues, charge groups with positive charge) used to control water solubility and their affinity for target cells [225,226]. For instance, synthetic porphyrins may be transformed into cationic entities through the insertion of positively charged substituents in the peripheral positions of the macrocycle which may largely affect the kinetics and extent of binding with microbial cells [44,100,103]. The charge on a photosensitizing molecule plays an important role in its biological properties. Cationic *meso*-substituted porphyrins have been proved to be more efficient and more photostable in water disinfection than other cationic PS as methylene blue (phenothiazinium dye) and rose bengal (xanthene dye) [227].

The nature and distribution of some functional groups in the molecule make it hydrophobic, hydrophilic or amphiphilic. Information about these properties is important because they affect, in a crucial way, the photophysical parameters and the efficacy of the PS [228]. A chemical compound is amphiphilic if possesses both hydrophilic and hydrophobic characteristics. In the transport system of cell membranes, the lipid bilayer of the membrane allows for passive transport of hydrophobic molecules. This means that molecules that repel water may diffuse across the cellular membrane without the need for an active transport system, like that of adenosine 5′-triphosphate. Therefore, the hydrophobic PS can more easily diffuse across the cell membrane and improve the efficiency of photodynamic effect. But as mentioned, the PS must be in solution, so it needs to be hydrophilic. Therefore, the ideal PS must have both hydrophilic and hydrophobic properties, making it amphiphilic. There are some factors which increase the amphiphilic character of the porphyrins such as the asymmetric charge distribution at their peripheral positions, and charges combined in different patterns with highly lipophilic groups (e.g., trifluoromethyl groups) [229–235]. The increase in the amphiphilic character of the PS seems to enhance its affinity for microorganisms which helps a better accumulation in the cells/particles [231,235,236] accompanied by an increase in the photocytotoxic activity [233]. Besides lipophilicity properties, other important parameters in the make-up of the PS must be considered. The degree of ionization, electric charge, molecular size, non-specific protein binding are same of the other factors which influence the photodynamic therapy response [237].

### 3.2. Photodymamic Therapy and Its Clinic Applications

At the beginning of its history, the first photochemotherapeutic approach was applied in medicine in 1903 by Albert Jesionek and Hermann von Tappeiner to the treatment of patients with malignant skin lesions [238]. However, since the middle of the last century, photochemotherapy applied to microorganisms was forgotten mainly because of the discovery and massive production of antibiotics [202]. Nowadays, photochemotherapy applications are again found in medicine, namely in the treatment of tumours. Photodynamic therapy (PDT) is the name commonly given to photochemotherapy of cancer [200]. In PDT, the administered photosensitizing compound selectively accumulates in the target cells and local irradiation is employed on the lesion (visible light, normally laser light delivered by optical fibers) [201,239,240]. The combination of two nontoxic elements, i.e. drug and light in the presence of molecular oxygen in the surrounding medium results in the selective destruction of tissue [239] through very localized cytotoxic effect [201].

Since the 1990’s, PDT has been successfully employed in the treatment of many tumors, including skin cancer, oral cavity cancer, bronchial cancer, esophageal cancer, bladder cancer, head and neck tumors in addition to nonmalignant diseases [241]. Besides cancer treatment, a very successful PDT application (FDA approved) has been in ophthalmology, to treat age-related macular degeneration [242].

Although the main use of PDT has focused on cancer treatment, the worldwide emergence of antibiotic resistance amongst pathogenic bacteria has led to a major research effort to find alternative antibacterial therapeutics [243] to which, it is hypothesized, microorganisms will not easily be able to develop resistance.

Currently, photodynamic antimicrobial chemotherapy (PACT) is receiving considerable attention for its potentialities as a new form of antimicrobial treatment [44,203,206]. All studies that have examined the killing of antibiotic resistant bacteria by the combination of PS and light, have found them to be equally as susceptible as their native counterparts [203,204,244]. Studies carried out with porphyrin derivatives pointed out to the great potentialities of this type of compounds for biomedical applications [196,228]. Recent studies, in particular, have demonstrated that PACT can be effective in the selective inactivation of microorganisms and it can become a potential alternative for the treatment and eradication of microbial infections [202,245]. Because the delivery of visible light is almost by definition a localized process, PACT for infections is likely to be applied exclusively to localized disease, as opposed to systemic infections, such as bacteremia [202]. In contrast to PDT for cancer, where the PS is usually injected into the bloodstream and accumulates preferentially in the tumor cells, PACT for localized infections is thought to be carried out by local delivery of the PS into the infected area by several methods such as topical application, instillation, interstitial injection or aerosol delivery [202]. The key issues that need to be addressed considering the photoinactivation of microorganisms are the effectiveness of the treatment in destroying sufficient numbers of the disease-causing pathogens; effective selectivity of the PS for the microbes, thus avoiding an unacceptable degree of photodynamic damage to host tissue in the area of infection; and the avoidance of regrowth of the pathogens from a few survivors following the treatment [202]. One of the main applications of PACT in the clinical area consists in the sterilization of blood and blood products, preventing of viral contamination. Remarkably, the human immunodeficiency virus has been inactivated *in vitro* by PACT [207,246–250]. Further applications of PACT would be skin surface disinfection, decolonization of nasal MRSA and wound healing in the future [44,202,237,251]

### 3.3. Photodymamic Antimicrobial Therapy Application in the Environment

Currently, photodynamic antimicrobial therapy has been studied having in view not only its application to the clinical field, but also to the environmental area [204,227,232,233,235,236,244, 252–269] (Table 2). This approach has been considered as a possibility for use in water disinfection [227,270]. Traditional water disinfection methods use chlorine, chlorine dioxide, ozone and ultraviolet radiation and are very efficient against a large range of microorganisms [271]. However, those treatments involve high costs and difficulties of implementation at large scale due to operational, personnel qualification and logistic deficits [271]. Ultraviolet radiation and ozone treatment are very expensive options to apply to a larger scale and disinfectants form toxic by-products, being the chlorine’s by-products the most toxic ones [272]. Effectiveness of PACT was observed on the destruction of faecal bacteria [227,253,273–277], viruses [278,279] and helminths eggs [280] in environmental waters.

The extension of the photodynamic principle to a new environmentally-friendly technology can become economically viable if the PS is immobilized on a solid matrix in order to allow the photoinactivation process and subsequent retention of the PS, after photodynamic action, to avoid the release of the PS to the water output [277,281]. As a consequence, some studies have developed PS immobilized on solid supports and the photodynamic inactivation (PI) against faecal bacteria was tested [232,275,277,281,282]. Bonnett *et al.* used a phthalocyanine immobilized on a polymeric membrane of chitosan in a model reactor of water disinfection [281]. They used an *E. coli* suspension of 10^5^ cell mL^−1^ representing the significant levels of water contamination. Also, when the dyed membrane was stored in the dark for nine months, the photodynamic action was still detectable, demonstrating the thermodynamic stability of the PS system. They concluded that with that model system, the photoinactivation with immobilized PS can be used to lower microbial levels in water flow systems and that might also have applications in water detoxification [281]. The same group also immobilized zinc phthalocyanines in a silicate matrix to test their photobactericidal properties on *E. coli* in model aqueous media. Although they obtained moderate PI, they concluded that phthalocyanines can be immobilized successfully in a silicate matrix and also used for photodisinfection of microbially polluted waters [232,275,277,281,282]. Regenerated cellulose impregnated with 5,10,15,20-tetrakis(1-methylpyridinium-4-yl)porphyrin tetra-*p*-tosylate showed photobactericidal activity against *S. aureus*, *E. coli*, *Proteus vulgaris* and *Bacillus subtilis* [275]. Two mono-hydroxyl zinc-porphyrin derivatives were immobilized on poly(methyl methacrylate), with respectively anionic and cationic net charge. The cationic derivative immobilized on poly(methyl methacrylate) polymer showed to have effective inhibitory effect in the photoinactivation of *Deinococcus radiodurans* [283]. Krouit *et al.* showed efficient photoinactivation of Gram-positive and Gram-negative bacterial strains by cellulose films with immobilized porphyrin derivatives [284–286]. Presently, new inorganic-organic hybrids (‘smart’ materials) based on tricationic porphyrin derivatives are also used at the photoinactivation of a large spectrum of microorganisms [287]. Preliminary results obtained with a tricationic porphyrin derivative immobilized in nanoparticles showed that inorganic-organic hybrids present antimicrobial activity and allows the recovery and recycling of the photosensitizing agent [287–289]. One of the tested inorganic-organic hybrids was able to inactivate Gram-positive, Gram-negative and T4-like bacteriophage to the limits of detection (up to 7 logs of inactivation) as the PS did in the unbound form [288,289]. Besides, Jiménez-Hernández *et al*. used as sensitizers, Ru(II) polypyridyl complexes grafted to polymer in a homemade microreactor with a solar simulator source for laboratory-scale water disinfection assays using a water sample containing *E. coli* and *Enterococcus faecalis* (2 × 10^3^ CFU mL^−1^). They concluded that photodisinfection with visible light was significant against both microorganisms [277].

### 3.4. Photodynamic Antimicrobial Therapy Application in Fish Farm Plants

Although just a few studies have been conducted in this field (Table 2), preliminary results obtained at both laboratory level and pilot station suggest that the photochemical technique, using porphyrin derivatives as PS, has a great potential also for the disinfection of fish farming plant waters [100,290]. These studies showed that cell cultures of Gram-positive bacteria (e.g., meticillin-resistant *S. aureus*), Gram-negative bacteria (e.g., *E. coli*), fungal (e.g., *C. albicans*) and fungal-like pathogens (e.g., *Saprolegnia* spp.) and parasitic protozoa (e.g., *Acanthamoeba palestinensis*) showed a 5–6 log decrease in the microbial population after 10 minutes of irradiation with low light intensities (ca. 50 mW cm^−2^) in the presence of micromolar PS doses [290]. Magaraggia *et al*. have also shown that a micromolar concentration of a porphyrinic PS promoted the cure of saprolegniosis in trout-farming pools containing naturally or artificially *Saprolegnia* infected fish (inactivation of 6–7 logs) without perilesional damage of the fish. A stock of fish were transferred to a 1,000 L tank and, after acclimatization, skin fish was infected by scraping dorsal trout epidermis and inoculated with *Saprolegnia* by direct contact of the lesions with mycelium wads. The infected group was dark incubated with 0.6 mg L^−1^ for 10 min in an 80–150 L pool and irradiated for 1 h kept in a closed circuit and recirculated by a motor-driven pump. The irradiation was performed by using the 400–800 nm wavelength interval emitted from two 100 W incandescent filament lamps and the water temperature was kept at 13 °C throughout the light exposure. The treatment was daily repeated for six consecutive days. After each treatment repetition, fish were moved to a 1,000 L tank. The onset of the infection in healthy fish was reduced about 50%. Recurrence of the saprolegniosis in the *Saprolegnia* infected sites or in others sites of the fish was not observed. The trout set with spontaneous infection by *Saprolegnia* a complete remission of the infection was induced within one week. The same micromolar concentrations exhibited also higher photosensitizing activity over meticillin-resistant *S. aureus* and *E. coli* (up 7 logs decrease) [100]. The antimicrobial effects of PDT were also demonstrated for *V. vulnificus* that frequently infects fish farming water [291]. Similarly, ten bacterial species (*V. anguilarum, V. parahaemolyticus*, *Photobacterium damselae* subsp. *damselae*, *Photobacterium damselae* subsp. *piscicida*, *A. salmonicida*, *E. coli*, *Enterobacter*, *S. aureus*, *E. faecalis*, *Pseudomonas* sp.) isolated from a fish farming plant waters were effectively inactivated (up to 7 logs) *in vitro* with cationic porphyrins, at micromolar PS doses, after 90–270 minutes of irradiation with a very low light intensity of 4 mW cm^−2^ [292], showing that photodynamic therapy can be used to photoinactivate fish bacterial pathogens in fish farm waters even during dark days of winter time. In these experiments fifty milliliters of bacterial suspensions from bacterial cultures (~10^8^ cells mL^−1^) were diluted ten-fold in phosphate buffered saline to a final concentration of ~ 10^7^ colony forming units mL^−1^ and exposed, in 600 mL glass beakers, to the PS under the white light. Bacterial inactivation was evaluated by counting the number of colonies, by pour plating method, in the exposed samples.

Irradiation of fish farming waters by solar light, which penetrates deeply into the water column, thereby allowing the uniform illumination of large volumes [293] makes this technology inexpensive since it is based on the use of low cost visible light sources.

The promising results of PACT on a large range of microorganisms, Gram-positive and Gram-negative bacteria including multidrug-resistant strains, bacterial spores, virus, bacteriophages, yeasts and helminths eggs [233,243,255,256,267,273,274,276,279,294–299] and the knowledge that the porphyrins’ mode of action make the selection of photoresistant strains very unlikely [237,300], suggest that this principle can be applied to photodecontamination of fish farming plants, in order to destroy pathogenic microorganisms. To implement this technology in fish farming plants some studies will be need to be carried out, namely pertaining to the determination of the stability of the new hybrid-porphyrin conjugates under visible light irradiation conditions. Moreover, there are no studies on the impact that this procedure might have on the total microbial community structure after treatment.

### 3.5. Advantages of Photodynamic Antimicrobial Chemotherapy over other Treatments in the Environment

The main favorable aspects of PACT for environmental use are the following [44,251]: (1) a broad spectrum of action: the PS inactivates efficiently bacteria, viruses, fungi, and parasites in both the dormant and vegetative states contrarily to chemotherapy and phage therapy; (2) an efficient phototoxic activity against both wild and antibiotic-resistant microbial strains; (3) the lack of selection of photoresistant microbial species; (4) a low mutagenic potential; (5) a high selectivity in the killing of pathogens as compared with the main constituents of potential host tissues; (6) a high selectivity in space and time: the microsecond short lifetime and high reactivity of singlet oxygen (the main pathway of PACT inactivation), restricts the photooxidative damage to the microenvironment of the site where it is generated to about 0.1 μm [194,195]; (7) the lack of generation of potentially dangerous or toxic by-products from photoinduced degradation of the photosensitizing agent; (8) cost-effective technology, as it is based on the use of visible light sources, as solar irradiation; (9) the possibility to reuse the immobilized PS which makes this technology less expensive and avoids its diffusion to the environment, preventing any risk of environmental contamination.

## Conclusions

The possibility to inactivate fish pathogenic microorganisms with phages and/or immobilized photosensitizers is outstanding, being the major advantage the efficient water disinfection degree obtained without risk to fishes or to the environment. The safety of the photodynamic therapy approach is increased as porphyrin derivatives apparently do not induce the selection of resistant microbial strains. Although, in some cases, phage therapy approach can lead to the selection of resistant bacteria, its occurrence and impact is not as so frequent and harmful as antibiotics chemotherapy.

The new approaches described in this review are intrinsically low cost compared to the chemical compound normally used in aquaculture systems and are conceived to be environmentally-friendly and to exhibit a high level of safety for various ecosystems, as well as for humans, animals and plants.

More studies are needed in order to gather a detailed understanding of phage-bacterium and of photosensitizer-microorganisms interactions in aquaculture systems subjected to different environmental pressure. A better understanding of phage therapy and of photodynamic therapy as adjuvant use of antibiotics and/or other disinfectant in fish farming plants also need to be further studied.

Each of the two techniques has its advantages but also its limitations. The application of phage and of photodynamic therapies must be based on a careful evaluation of each case, before replacing conventional approaches. Nevertheless, a good biocontrol management strategy might be the use of more than one technique in rotation to prevent resistance development. Alternatively, it might be valuable to apply both techniques to maximize protection against pathogenic microorganisms.

## Figures and Tables

**Table 1:** Use of bacteriophages to control pathogenic bacteria.

Reference	Phage	Bacteria	Treated fish/shellfish	Phage application	Effects
Barrow *et al*., 1998 [147]	Bacteriophage R isolated from domestic sewage	*E. coli H247 (O18:K1:H7)* (bacteremic)	Chickens and calves	Intramuscular (10^2^–10^6^ PFU) and intracranial (10^6^ PFU) inoculation of chickens; oral and intramuscular inoculation of calves with 3 × 10^10^ PFU)	Protection against morbidity and mortality
Biswas *et al*., 2002 [153]	*Enterococcus* phages ENB6 and C33 isolated from raw sewage	Vancomycin-resistant *Enterococcus faecium* (agent of VRE bacteremia)	BALB/c mice	Intraperitoneal injection of 3 × 10^8^ PFU	Complete rescue of bacteraemia in 48 hours
Bogovazova *et al*., 1991 [156]	*Klebsiella pneumoniae* bacteriophage	*Klebsiella*	BALB/c mice	Intraperitoneal, intravenous or intranasal administration	Rescue of generalized *Klebsiella* infection
Cao *et al*., 2000 [155]	*Helicobacter pylori* M13 recombinant phage	*Helicobacter pylori*	BALB/c mice	Oral administration of 10^7^ PFU	Reduction of stomach colonization by *Helicobacter pylori*
Fiorentin *et al*., 2005 [157]	*Salmonella enteritidis* lytic phages CNPSA 1, CNPSA 3, CNPSA 4	*Salmonella enteritidis*	Chicken cuts (thighs and drumsticks)	Immersion in 10^9^ CFU mL^−1^ bacteriophage suspensions	Reduction of *Salmonella enteritidis* counts in treated chicken cuts
Flaherty *et al*., 2000 [165]	*Xanthomonas campestris* pv. *vesicatoria* specific H-mutant bacteriophages	*Xanthomonas campestris* pv. *vesicatoria*	“Sunbeam” tomato *Lycopersicon esculentum*	Foliar applications of 10^8^ PFU mL^−1^ phage suspensions	Reduction of bacterial spots and increase in fruit weight
Goode *et al*., 2003 [163]	*Salmonella enteritidis* phage types P125589, phage 29C and transducing lambdoid phage P22, HTint, isolated on *S. enterica* serovar Enteritidis from sewage; *Campylobacter jejuni* phage 12673	*Salmonella enterica* serovar Enteritidis and *Campylobacter jejuni*	Chicken skin	Surface spreading with 10^3^ PFU cm^−2^	Reduction by 2 log units in bacterial abundance over 48 hours
Hagens *et al*., 2004 [149]	Genetically engineered non-replicating, non-lytic filamentous phage Pf3R obtained from phage Pt1 isolated from river water using PAO1 as the host	*Pseudomonas aeruginosa*	BALB/c mice	Intraperitoneal inoculation with 10^6^–10^8^ PFU	Higher survival rate and reduced inflammatory response after 12–24 hours
Huff *et al*., 2005 [164]	*Escherichia coli* phages SPR02 and DAF6	*Escherichia coli* isolated from poultry	Broiler chickens	Injection in the air sac with 10^4^ or 10^8^ PFU mL^−1^ phage suspensions and bird spraying with phage suspensions	Decreased bird mortality
Imbeault *et al*., 2006 [175]	HER 110	*Aeromonas salmonicida HER 1107*	Brook trout *Salvelinus fontinalis*	Addition to aquarium water of stock bacteriophage suspensions 10^9^ PFU mL^−1^	The onset of furunculosis in brook trout was delayed by 7 days
Jado *et al*., 2003 [154]	Phage-coded lysins (enzybiotic): Pal amidase and/or Cpl-1 lysozyme	Antibiotic-resistant *Streptococcus pneumoniae* 541, serotype 6B	BALB/c mice	Intraperitoneal injection of 1 mg mL^−1^ (110 000 U mg^−1^) enzyme solutions	Rescue of bacteraemia and prevention of death in 72 hours
Karunasa gar *et al*., 2007 [130]	Siphoviridae isolated from from oyster tissue and from shrimp hatchery water	*Vibrio harveyi*	Shrimp larvae *Penaeus monodon*	Ammendment of water in hatchery tanks with bacteriophage suspension (10^6^ PFU mL^−1^)	Improved larval survival
Leverentez *et al*., 2001 [162]	*Salmonella* – specific phages	*Salmonella enteritidis*	Fresh-cut fruit (melons and apples)	Direct application of 5 × 10^7^ PFU mL^−1^ phage suspension on fruit slices	Reduction of *Salmonella* concentration by 2.5–3.5 logs on melon but not on apple
Matsuzaki *et al*., 2003 [151]	Bacteriophage fMR11	*Staphylococcus aureus*	BALB/c mice	Intraperitoneal inoculation with phage suspension	Higher survival rate and bacterial eradication in 1 and 7 days
Nakai and Park, 2002 [12]	Siphoviridae isolated from diseased fish and sea water in fish culture cages.	*Lactococcus garvieae*, formerly *Enterococcus seriolicida*	Yellowtail *Seliora quinqueradiata* and Ayu *Plecoglossus altivelis*	Oral administration of phage-impregnated feed or intraperitoneal injection	Protective/curative effect (increase in the survival rate)
Nakai *et al*., 1999 [15]	Siphoviridae isolated from diseased fish and sea water in fish culture cages.	*Lactococcus garvieae*, formerly *Enterococcus seriolicida*	Yellowtail *Seliora quinqueradiata*	Oral administration of phage-impregnated feed or intraperitoneal injection	Protective/curative effect (increase in the survival rate)
Park and Nakai, 2003 [174]	PPp-W4 (Podoviridae) PPpW-3 (Myoviridae)	*Pseudomonas plecoglossicida*	Ayu *Plecoglossus altivelis*	Oral administration of phage-impregnated feed (10^7^ PFU/fish).	Reduced infection and increased fish survival
Park *et al*., 2000 [27]	Myoviridae and Podoviridae isolated from diseased ayu and the rearing pond water	*Pseudomonas plecoglossicida*	Ayu *Plecoglossus altivelis*	Oral administration of phage-impregnated feed	Protection against experimental infection
Toro *et al.*, 2005 [158]	*Salmonella* – specific bacteriophages	*Salmonella typhimurium*	Chicken	Oral administration	Reduction in *Salmonella* counts in cecum and ileum treated chickens
Verner-Jeffreys *et al.*, 2007 [176]	*Aeromonas salmonicida* phages O, R and B	*Aeromonas salmonicida* subsp. *salmonicida*	Atlantic salmon *Salmo salar*	Injection (1.9 × 10^8^ PFU/fish), oral administration (1.88 × 10^5^ PFU g^−1^) and bath (1.04 × 10^5^ PFU mL^−1^)	Lower rate mortality but similar absolute mortality. No protection was offered by any of the bacteriophage treatments.
Vinod *et al.*, 2006 [129]	Siphoviridae	*Vibrio harveyi*	Shrimp larvae *Penaeus monodon*	*In vitro* ammendment with bacteriophage suspension (10^9^ PFU mL^−1^)	Improved larval survival
Watanabe *et al.*, 2007 [150]	Phage strain KPP10 isolated from a highly polluted river using *P. aeruginosa* strain PA20 as the host.	*Pseudomonas aeruginosa* strain D4 (agent of gut-derived sepsis)	ICR mice	Intraperitoneal inoculation with 10^10^ PFU	Higher survival rate and reduced inflammatory response after 24 hours
Wills *et al.*, 2005 [152]	Bacteriophage LS2a	*Staphylococcus aureus* strain 2698 (abscess forming)	New Zealand White rabbits	Subcutaneous injection with 2 × 10^9^ PFU	Prevention of abscess formation

**Table 2:** Use of photosensitizers and light to inactivate pathogenic microorganisms.

Reference	Microorganisms	Photosensitizer (PS)	Concentration of PS	Irradiation time	Type of light	Light dose	Fluence rate	Cell concentration
Alouini *et al.*, 2001 [280]	Helminth eggs	Cationic *meso*-substituted porphyrin, tetra-(4-*N-*methylpyridyl) porphin tetratosylate (T4MPYP)	10 μM	30 minutes	Visible light		0.5 W cm^−2^	15–20 cell mL^−1^
Alves *et al.*, 2008 [273]	Recombinant bioluminescent *Escherichia coli (E. coli)*	Three cationic *meso-*substituted porphyrin derivatives	0.5 μM, 1μM and 5 μM	0–270 minutes	Artificial white light/sunlight	64.8 J cm^−2^/1004.4 J cm^−2^	40 W cm^−2^/~620 W cm^−2^	10^7^ CFU mL^−1^
Alves *et al.*, 2009 [274]	*E. coli* and *Enterococcus faecalis (E. faecalis)*	Seven cationic porphyrins differing in *meso-*substituent groups, charge number and charge distribution	0.5 μM, 1 μM and 5 μM	0–270 minutes	White light	64.8 J cm^−2^	40 W cm^−2^	10^7^ CFU mL^−1^
Banfi *et al.*, 2006 [230]	*E. coli*, *Pseudomonas aeruginosa* and *Staphylococcus aureus (S. aureus)*	Three tetracationic porphyrins, a dicationic porphyrin and a neutral porphyrin	0.4–60 μM	30–60 minutes	Visible light	266 J cm^−2^		10^8^ CFU mL^−1^
Caminos and Durantini, 2006 [232]	*E. coli* immobilized on agar surfaces	5,10,15-tris[4-(3-N,N,N-trimethylammoniumpropoxy) phenyl]-20-(4-trifluoromethylphenyl)-porphyrin iodide and 5,10,15,20-tetra(4-N,N,N-trimethylammoniumphenyl) porphyrin p-tosylate	0–14 nmol	0–180 minutes	Visible light (a projector or midday sun)		90 mW cm^−2^	small colonies on agar surfaces
Carre *et al.*, 1999 [252]	*Saccharomyces cerevisiae*	Neutral *meso-*arylglycosylporphyrins	10^−5^ M	10–120 minutes	Visible light (150 W tungsten lamps)		500 W m^−2^	10^7^ CFU mL^−1^
Carvalho *et al.*, 2007 [253]	Faecal coliforms	Two sets of neutral and cationic porphyrins	5 μM	270 minutes	White light (9 mW cm^−2^)			
Cormick *et al.*, 2009 [254]	*Candida albicans (C. albicans)*strain PC31	5-(4-trifluorophenyl)-10,15,20-tris(4-trimethylammoniumphenyl) porphyrin iodide (TFAP^3+^); 5,10,15,20-tetra(4-N,N,N-trimethylammoniumphenyl) porphyrin p-tosylate (TMAP^4+^); 5,10,15,20-tetra(4-sulphonatophenyl) porphyrin (TPPS4 ) sodium salt	1 μM–5 μM	240 minutes	Visible light (350–800 nm)		90 mW cm^−2^	10^6^–10^8^ CFU mL^−1^
Demidova and Hamblin, 2005 [255]	*E. coli, S. aureus*, *C. albicans*	Rose bengal, toluidine blue O, and a poly-L-lysine chlorin(e6) conjugate (pL-ce6)	2–3.3 mM		Visible light (noncoherent light source with interchangeable fiber bundles)	0 to 200 J cm^−2^	50 to 400 mW cm^−2^	10^7^–10^8^ CFU mL^−1^
Demidova and Hamblin, 2005 [256]	*Bacillus atrophaeus* (ATCC 9372), *B. cereus* (ATCC14579), *B. megaterium* (ATCC14581), *B. thuringiensis* (ATCC 33740) and *B. subtilis* (ATCC 6051)	Rose bengal, toluidine blue O, methylene blue, new methylene blue N (zinc chloride double salt; NMBN), 1,9-dimethylmethylene blue chloride (DMMB), 5-phenyl-10,15,20-tris(N-methyl-4-pyridyl-)porphyrin chloride [TriP(4)], poly-L-lysine chlorin(e6) conjugate, benzoporphyrin derivative	5μM–1.600 μM	180 minutes	Visible light (noncoherent light source with interchangeable fiber bundles)	0 to 200 J cm^−2^	200 to 400 mW cm^−2^	10^7^ CFU mL^−1^
Drábková *et al.*, 2007 [257]	Cyanobacteria	Phthalocyanines, tetraphenol porphyrine, methylene blue	0.001–5 mg L^−1^	60 minutes	White ligh (5000 lx under fluorescent tubes)		5000 lx under fluorescent tubes	10^5^–10^6^ CFU mL^−1^
Ehrenberg *et al.*, 1993 [258]	*S. aureus* and *E. coli*	Mg and Zn-tetrabenzoporphyrin						
Foschi *et al.*, 2007 [259]	*E. faecalis* (ATCC 29212)	Methylene blue	16.75 mM	10 minutes	Diode laser	60 J cm^−2^	100 mW cm^−2^	10^9^ CFU mL^−1^
Gábor *et al.*, 2001 [260]	*E. coli* and *Enterococcus hirae*	Exogenous and endogenous porphyrin derivatives	1.2 × 10^−6^–4 × 10^−3^ M	120 minutes	White ligh (halogen lamp)	0.08–0.25 W cm^−2^	10^7^ CFU mL^−1^	
Grinholc *et al.*, 2008 [261]	40 methicillinresistant *S. aureus* (MRSA) and 40 methicillin sensitive *S. aureus* (MSSA) strains; and also one reference strain of *S. aureus* (ATCC 25904)	Protoporphyrin diarginate (PPArg2)	25 μM		White ligh (624 nm)	12 J cm^−2^		
Jemli *et al.*, 2002 [227]	Fecal coliforms	Rose Bengal, methylene blue, *meso*-substituted cationic porphyrin	1μM, 5μM, 10μM	60 minutes	Sunlight	234 μM m^−2^ s^−1^	1235 mW cm^−2^	
Lazzeri *et al.*, 2004 [236]	*E. coli*	Asymmetric *meso*-substituted cationic porphyrins: 5,10-di(4-methylphenyl)-15,20-di(4-trimethylammoniumphenyl)porphyrin iodide 1 and 5-(4-trifluorophenyl)-10,15,20-tris(4-trimethylammoniumphenyl)porphyrin iodide 2 and its metal complex with Pd(II) 3 and a non cationic sensitizer 5-(4-carboxyphenyl)-10,15,20-tris(4-methylphenyl)porphyrin 4	10 μM	30 minutes	Withe ligh (slide projector equipped with a 150 W lamp)		90 mW cm^−2^	10^6^ CFU mL^−1^
Maisch *et al.*, 2005 [262]	Two MRSA strains, one MSSA strain, one methicillin-resistant *Staphylococcus epidermidis* strain, one *E. coli* strain	Porphyrin-based photosensitizers (CTP1, XF70, and XF73)	0–10 μM	15 minutes	Visible light (incoherent light source, UV236; 380 to 480 nm)	13.7 J cm^−2^	15.2 mW cm^−2^	10^8^–10^9^ CFU mL^−1^
Merchat *et al.*, 1996 [263]	*Vibrio anguillarum E. coli Enterococcus seriolicida*	Two *meso*-substituted cationic porphyrins: tetra(4N-methyl-pyridyl) porphine tetraiodide and tetra(4N,N,N-trimethyl-anilinium) porphine, and negatively charged *meso-*substituted porphyrin, tetra(4-sulphonatophenyl)porphine	10 μg mL^−1^	0–30 minutes	White light (four 250 W tungsten lamps)		6 mW cm^−2^	10^8^ CFU mL^−1^
Merchat *et al.* (b), 1996 [264]	*Vibrio anguillarum E. coli Enterococcus seriolicida*	*meso*-tetra (4-N-methyl-pyridyl) porphine tetraiodide, T4(4-N-MePy)P; *meso*-tetra (3-N-methyl-pyridyl)porphine tetrachloride, 1"4(3-N-MePy) P; tri(4-N-methyl-pyridyl) monophenylporphine Iritosylate, T3(4-N-MePy) PhP; di(N-methyl-4-pyridyl)diphenylporphine dichloride (D(4-N-MePy) Ph2P	8.4 μM	0–30 minutes	White ligh (250 W quartztungsten lamps)		6 mW cm^−2^	
Nitzan and Ashkenazi, 2001 [266]	*Acinetobacter baumannii* and *Escherichia coli* B	Cationic TMPyP	29.4 mmol L^−1^ (p), 3.7 mmol L^−1^ (}), 1.83 mmol L^−1^ (F), and 0.73 mmol L^−1^ (h)		Blue, green and red light		140–150 mW cm^−2^	10^9^ CFU mL^−1^
Nitzan *et al.*, 1998 [269]	*Acinetobacter baumannii*	Deuteroporphyrin (Dp) and polymyxin nonapeptide (PMNP) ; Cd-texaphyrin (Cd-Tx) in the presence of PMNP; cationic photosensitizer tetramethylpyridyl porphine (TMPyP); anionic photosensitizer tetra-sulfonatophenyl porphine (TPPS4)	Deuteroporphyrin (Dp) at a concentration of 34 Ixmoles I ~ and polymyxin nonapeptide (PMNP) at a concentration of 200 txmoles 1 ~	0–210 minutes	White ligh (unfiltered tungsten lamps)		140 W m^−2^	10^8^ CFU mL^−1^
Oliveira *et al.*, 2009 [267]	*Bacillus cereus* endospores and vegetative cells	Neutral and cationic porphyrin derivatives, and phenothiazinium dye toluidine blue O and 10,15,20-tris(1-methylpyridinium-4-yl)-5-(phenyl)porphyrin tri-iodide (Tri-Py+-Me-Ph, tricationic	10 and 60 μM	4 and 10 minutes for endospores and for 15 minutes for vegetative cells	White light (400–800 nm)	152.1 J cm^−2^ (maximum dosis)	1690 W m^−2^	10^6^–10^7^ CFU mL^−1^
Spesia *et al.*, 2005 [235]	*E. coli*	Meso-substituted cationic porphyrins, 5-[4-(trimethylammonium)phenyl]-10,15,20-tris(2,4,6-trimethoxyphenyl)porphyrin iodide 1, 5,10-di(4-methylphenyl)-15,20-di(4-trimethylammoniumphenyl)porphyrin iodide 2 and 5-(4-trifluorophenyl)-10,15,20-tris(4-trimethylammoniumphenyl)porphyrin iodide 3	10 μM	5 minutes	Visible light		0.68, 2.60 and 90 mW cm^−2^	10^6^ CFU mL^−1^
Tang *et al.*, 2007 [268]	*S. aureus* (ATCC 25923), *E. coli* (ATCC 25922), a clinical isolate of MRSA, and a clinical isolate of ESBL-producing *E. coli*	Toluidine blue O and poly-l-lysine chlorin(e6) conjugate (pL-ce6)	4–8 μM	30 minutes	Red light	10–30 J cm^−2^	400 W	10^8^ CFU mL^−1^
Wainwright *et al.*, 1998 [204]	MRSA strains	Phenothiazinium dyes						
Wilson and Yianni, 1995 [244]	MRSA strain	Toluidine blue	1.6–12.5 μg mL^−1^	1 minute	Low power helium/neon laser	0.5–2.1 J cm^−2^	35 mW	10^10^ CFU mL^−1^
